# Supplementary material for: Design and realization of compressor data abnormality safety monitoring and inducement traceability expert system
Source: PLoS One. 2025 Jan 16;20(1):e0315917. doi: 10.1371/journal.pone.0315917 (PMC11737859; doi:10.1371/journal.pone.0315917)
Supplement: Attachment — Submitted filename: Response to reviewer revised.docx [file pone.0315917.s005.docx]

Dear Editor:

Thank you very much for giving us an opportunity to revise our manuscript, we appreciate you and the reviewers very much for their positive and constructive comments and suggestions on our manuscript entitled “Design and realization of compressor data abnormality safety monitoring and inducement traceability expert system”.

We carefully studied the reviewers' comments and made revisions in a comparatively revised manner. We have tried our best to revise our manuscript according to the comments. Attached please find the revised version, which we would like to submit for your kind consideration.

We would like to express our great appreciation to you and reviewers for reviewing and evaluating our manuscript. Looking forward to hearing from you.

Thank you and best regards.

Yours sincerely,

Yuan Wang

Corresponding author:

Name: Shaolin Hu

E-mail:hfkth@126.com

Dear reviewers:

Thank you for your excellent and insightful series of remarks. We have processed these comments you raised and made the modifications in the form of comparative revisions. We hope that the revision is acceptable and look forward to hearing from you soon.

Thank you and best regards.

Yours sincerely,

Yuan Wang

Corresponding author:

Name: Shaolin Hu

E-mail:hfkth@126.com

**Reviewer #1:**

**Q****1.** The authors need to revise the numerical enumeration of Sections in the manuscript. For example, the numbering of Section 2 is repeated twice.

**Response:** Thank you for your detailed review and valuable feedback on the manuscript. I have carefully reviewed the manuscript and made corresponding modifications to address the issue of duplicate chapter numbers that you pointed out, such as Section 2 being duplicated twice..

**Q2.** While abbreviations like PCA-SPE and CNN are common in the field, providing a brief explanation of each in the abstract would help readers unfamiliar with these terms.

**Response:** Thank you for your careful review and valuable suggestions on my manuscript. I completely agree with your point about providing brief explanations of abbreviations in the abstract (such as PCA-SPE and CNN). Short explanations can indeed help readers who are not familiar with these terms better understand the content of the article.

I have made the following modifications to the abstract: When PCA-SPE and CNN first appear in the abstract, their full names and brief explanations (principal component analysis squared prediction error) and (convolutional neural network) are provided.

**Q3.**  I would recommend combining PCA-SPE with other dimensionality reduction techniques or neural networks) which could possibly increase robustness in different operational conditions.

**Response:** Thank you for your suggestion. After careful consideration and analysis, I have the following opinions on this suggestion:

1. Information redundancy and increased complexity: PCA is a linear dimensionality reduction method that, when combined with highly nonlinear techniques such as neural networks, may result in mismatched linear features, thereby increasing the complexity and tuning difficulty of the model. Especially in neural networks, the linear features generated by PCA may not effectively capture nonlinear features, which may instead introduce redundant information.
2. Computing resources and time overhead: The training process of neural networks itself requires a significant amount of computing resources and time. If combined with PCA-SPE, although PCA can reduce data dimensionality, it may further increase the training time and resource consumption of the entire model, especially when dealing with large-scale datasets.
3. Model interpretability issue: Although the results of PCA have some interpretability (each principal component represents certain linear features), the nonlinearity and black box nature of neural networks make interpretation difficult, which may reduce the interpretability of the model and affect the trust of experts and users in the results.
4. Risk of excessive reduction of information: Although PCA aims to preserve the most important features, continuing to use other dimensionality reduction methods after PCA may further reduce key information in the data. Even if each dimension reduction step removes redundant information, it may ultimately lead to excessive reduction of useful information, which has a negative impact on subsequent classification tasks.

Therefore, based on the above points, I have decided to maintain the original PCA-SPE method to avoid issues such as information redundancy, increased complexity, and decreased interpretability. However, your suggestion inspired me to explore in future research how to balance the combination of linear and nonlinear features while preserving model simplicity.

Thank you again for your valuable feedback. Looking forward to your further feedback!

**Q4.**  I would highly suggest the authors to add a Related Works section that provides information on the previous researches that were conducted in the same field and identify ways that this research has contributed to the previous ones. Perhaps, whether the research conducted was an idea obtained from previous work's future directions.

**Response:** Thank you for your detailed review of my manuscript and valuable suggestions. I fully agree with your suggestion to add relevant works as an effective way to enhance the background information and academic contribution of the paper.

I have made the following modifications in the manuscript:

New references have been added to the preface, which provides a detailed introduction to previous work related to my research field, covering the latest research findings and key contributions. A summary was made of the core methods, results, and findings from these previous studies, and the differences and extensions of this study from these works were clearly identified.

**Q5.** The authors should provide a more clear and high resolution image for Figure 14. The text is barely visible even when the image is largely scaled. Same goes for Figure 6.

**Response:** Thank you for pointing out the quality issues with Figures 14 and Figures 6. I have replaced the higher resolution images for Figure 14 and Figure 6 to ensure that the text and image details are clearly visible.

**Q6.** Since the method is intended for real-time fault detection, discussing how it deals with noisy, incomplete, or anomalous data in real-world settings would be valuable.

**Response:** Thank you for the reviewer's suggestions. Our research mainly focuses on the actual operating data and corresponding fault cases of the compressor at Gaoling Station provided by China Petroleum West East Gas Pipeline Co., Ltd. I attach great importance to your suggestion on how to handle noise, incomplete or abnormal data in real environments using this method. In each stage of data processing, I applied fault-tolerant filtering algorithms for data processing, and used residual generation and data anomaly monitoring algorithms based on fault-tolerant filtering to monitor anomalies in each dimension of the data. This method demonstrates adaptability and robustness in practical complex environments. Thank you again for your valuable feedback. Looking forward to further feedback!

**Q7.** I would suggest the authors to provide more details on the real-time updating mechanism of the dynamic knowledge graph. Specifically, explain how the system handles large, incremental updates and maintains accuracy without introducing delays or compromising system efficiency.

**Response:** Thank you for your suggestion. Regarding the process of using a graph database to store knowledge graphs and implementing iterative optimization, we have implemented the following steps:

1. Real time data reception: The system continuously receives instrument data and process data from the petrochemical DCS system to ensure the timeliness and integrity of the data.
2. Incremental data information processing: By combining incremental data, we can update the knowledge graph in real-time and achieve rapid iteration of knowledge.
3. PCA-SPE-CNN classifier: By constructing a PCA-SPE-CNN classifier, we classify faults in real-time monitored instrument data to ensure accurate identification of potential faults.
4. Combining knowledge graph with expert knowledge: We combine fault classification results with expert knowledge in the knowledge graph to promote knowledge updates and iterations, achieving a more efficient inference process.

The ultimate goal of this method is to continuously improve the intelligence level of the knowledge graph while ensuring system accuracy and efficiency. Looking forward to your further guidance.

**Q8.** The authors should consider restructuring the sections so that there is a clear progression from data collection and processing to knowledge graph construction, and finally, to fault diagnosis and traceability.

**Response:** Thank you for your suggestion. We have made structural adjustments and look forward to your further guidance.

**Reviewer #2:**

**Q1.** It is strongly recommended that the authors either expand the introduction or add a new separate section reviewing related works in the field. This would help better substantiate the innovative aspects of the research and highlight how it addresses a specific gap, contributing to the existing body of knowledge. Attention should be paid to the fact that the references already included in this article are relatively limited and do not feature particularly research studies (i.e. from the last five years), constraining both the quality and the potential impact of the article.

**Response:** Thank you for your detailed review of my manuscript and valuable suggestions. I fully agree with your suggestion to add relevant works as an effective way to enhance the background information and academic contribution of the paper.

I have made the following modifications in the manuscript:

New references have been added to the preface, which provides a detailed introduction to previous work related to my research field, covering the latest research findings and key contributions. A summary was made of the core methods, results, and findings from these previous studies, and the differences and extensions of this study from these works were clearly identified.

**Q2.** The authors are strongly encouraged to revise the structure of the manuscript.
In particular, careful attention has to be paid to the proper numbering of the sections. For instance, Section 2 is attributed both to “CONSTRUCTION OF A RESIDUAL GENERATION AND DATA VARIANCE MONITORING ALGORITHM BASED ON FAULT-TOLERANT FILTERING” and “CONSTRUCTION OF A DEEP LEARNING BASED SYSTEM FAULT CLASSIFIER”. Moreover, subsections should not include only a paragraph (i.e. subsection 4.4) or a Figure (i.e. subsection 4.6).

**Response:** Thank you for your suggestion. We have made structural adjustments and look forward to your further guidance.

**Q3.** Outlining potential directions for future research in the conclusions section, could enhance the impact of the research outcomes.

**Response:** Thank you for your in-depth review of our work. We have outlined the potential directions for future research in the conclusion section. Through the overview of these potential directions, we hope to provide inspiration for future research and lay the foundation for further practical applications.

**Q4.** Structure and English Language: The paper is written in appropriate English language according to the standards of the Journal, however the authors are strongly recommended to conduct some proofreading and spell-checking.

**Response:** Thank you for your meticulous review and feedback on our paper. We have conducted language proofreading.

**Reviewer #3:**

**Q1.** Data is not explained, descriptive stats about data is missing, shape of data, features in data, distribution of class etc.

**Response:** Thank you for your feedback on our paper. We have added a section on "Data Cause Analysis" in the revised version to provide a detailed description of the source, shape, characteristics, and other relevant information of the data.

**Q2.** Page 25 – “the convolution kernel is 1*3, the number of convolution kernels is 16, 16, 64, 64, 256, 256, the activation function is the function, and the pooling layer is the maximum pooling and average pooling.” What is meant by the activation function is the function”?

**Response:** Thank you for your valuable feedback. We will further clarify in the revised version that the description of "activation function" is a $relu$ function.

**Q3.** The introduction part is not explained well, no literature review.

**Response:** Thank you for your detailed review of my manuscript and valuable suggestions. I fully agree with your suggestion to add relevant works as an effective way to enhance the background information and academic contribution of the paper.

I have made the following modifications in the manuscript:

New references have been added to the preface, which provides a detailed introduction to previous work related to my research field, covering the latest research findings and key contributions. A summary was made of the core methods, results, and findings from these previous studies, and the differences and extensions of this study from these works were clearly identified.

**Q4.** hyperparameters not listed.

**Response:** Thank you for your valuable feedback on our paper. We have added a section on "Parameter tuning" in the revised version to provide a detailed list of hyperparameters used, including learning rate, batch size, and regularization parameters.

**Q5.** Future study directions that concentrate on different tracks have not been added by the writers for the benefit of upcoming scholars who wish to further the academics in this sector.

**Response:** Thank you for your in-depth review of our work. We have outlined the potential directions for future research in the conclusion section. Through the overview of these potential directions, we hope to provide inspiration for future research and lay the foundation for further practical applications.
