# Supplementary material for: Design and realization of compressor data abnormality safety monitoring and inducement traceability expert system
Source: PLoS One. 2025 Jan 16;20(1):e0315917. doi: 10.1371/journal.pone.0315917 (PMC11737859; doi:10.1371/journal.pone.0315917)
Supplement: Attachment — Submitted filename: Response to reviewer revised.docx [file pone.0315917.s006.docx]

Dear Editor:

Thank you very much for giving us an opportunity to revise our manuscript, we appreciate you and the reviewers very much for their positive and constructive comments and suggestions on our manuscript entitled “Design and realization of compressor data abnormality safety monitoring and inducement traceability expert system”.

We carefully studied the reviewers' comments and made revisions in a comparatively revised manner. We have tried our best to revise our manuscript according to the comments. Attached please find the revised version, which we would like to submit for your kind consideration.

We would like to express our great appreciation to you and reviewers for reviewing and evaluating our manuscript. Looking forward to hearing from you.

Thank you and best regards.

Yours sincerely,

Yuan Wang

Corresponding author:

Name: Shaolin Hu

E-mail:hfkth@126.com

Dear reviewers:

Thank you for your excellent and insightful series of remarks. We have processed these comments you raised and made the modifications in the form of comparative revisions. We hope that the revision is acceptable and look forward to hearing from you soon.

Thank you and best regards.

Yours sincerely,

Yuan Wang

Corresponding author:

Name: Shaolin Hu

E-mail:hfkth@126.com

**Reviewer #1:**

**Q****1.** The authors have done a well job in constructing the abstract as it is presented very effectively and summarizes the key contributions of the research

**Response:** Thank you for the reviewer's recognition of the abstract section. We are pleased that you believe the abstract effectively summarizes the main contributions of the research. Your affirmation has inspired us, and we appreciate your support and recognition of our work.

**Q2.** The authors have chosen 5 as the value for their default k-parameter in the sliding window method. Perhaps they should explain more as to why they have chosen 5?

**Response:** Thank you for your careful review and valuable suggestions on my manuscript. In our study, the choice of k=5 was based on the following considerations:

1. Choosing 5 is usually because it can provide a good balance in practical applications, effectively filtering out noise while preserving the overall trend of the data.

2. The size of the sliding window directly affects the computational complexity. A smaller window width can usually avoid excessive computational overhead while ensuring filtering effectiveness.

3. We chose k=5 as a reasonable value obtained through experimental evaluation. After multiple experiments, k=5 provides a good balance between the temporal correlation of data and computational efficiency.

**Q3.** The authors should explain more about the dataset that they have used for training and validation.

**Response:** Thank you for your suggestion. We have provided a more detailed explanation of the application of the dataset and algorithm. The residual generation and data anomaly monitoring algorithm based on fault-tolerant filtering is mainly used to detect the stationarity of each dimensional feature. The monitoring data used is individual data for each dimension and does not affect each other. The construction of classifier algorithms mainly considers the impact of twelve dimensional features on second-class faults, and the monitoring data is a comprehensive consideration of twelve dimensional data to capture the correlation between different features and improve the classification accuracy of the model.

Thank you for your suggestions. These supplements and explanations will help to better understand the structure of the dataset and the design ideas of the algorithm.

**Q4.** To ease off the comprehension factor for readers, the authors should try to add more simple termed notes for the complex mathematical formulas that are presented in the manuscript.

**Response:** Thank you for your detailed review of my manuscript and valuable suggestions. More annotations have been added to complex formulas in the text.

**Q5.** The authors should revise the citations and make sure that there is a consistent citation style throughout the manuscript.

**Response:** Thank you for your detailed review of my manuscript and valuable suggestions. Modifications have been made.

**Reviewer #4:**

**Q1.** The authors are highly recommended to re-enumerate the sections properly. For example, first Section should begin with 1, and not 0. Additionally, it should be in a high priority since it was already stated in previous revision to re-enumerate properly..

**Response:** Thank you for your detailed review of my manuscript and valuable suggestions. Modifications have been made.

**Q2.** There is very minimal information provided in terms of overview even for major section headings such as Section 2.

**Response:** Thank you for your suggestion. We have made revisions and look forward to your further guidance.

**Q3.** The authors are suggested to provide an overview for major headings such as Section 4 before straightly jumping to subsections, in this case, subsection 4.1.

**Response:** Thank you for your suggestion. We have made revisions.

**Q4.** Why is subsection 4.6 left empty and there is no content presented within it?

**Response:** Thank you for your feedback on our paper. We have made revisions.
